# Supplementary material for: Development of Good Manufacturing Practice-Compatible Isolation and Culture Methods for Human Olfactory Mucosa-Derived Mesenchymal Stromal Cells
Source: Int J Mol Sci. 2024 Jan 6;25(2):743. doi: 10.3390/ijms25020743 (PMC10815924; doi:10.3390/ijms25020743)
Supplement: Supplementary file 1 [file ijms-25-00743-s001.zip › OM-MSC - Antibiotic CS022 Batches 1-3.pdf]

Product Testing Unit SNBTS - Default
ELx808 reader
Lonza - WinKQCL Version 5.1.2

Performed By : F.MITCHELL	LAL Lot No.	: XL041KRP2Y	Exp : 02/04/2023	Time : 12:11:25
PYROGENT-5000 - Routine Test	Water Lot No.	: 0001098994	Exp : 10/03/2024	Date : 05/10/2022
Template : 051022/01	Endotoxin Lot No.	: 0001011300	Exp : 02/06/2025	ELx808
Temperature : 36.9°C - 37.0°C	Recon. Buffer Lot No.	: 0001019067	Exp : 01/07/2023	S/N 253635

Linear Regression : Correlation Coefficient = -0.997 Slope = -0.238 Y-Intercept = 2.820

PowerCurve : A = 2.8000 B = -0.2119 C = 0.0179 D = -0.0028 E = 0.0000

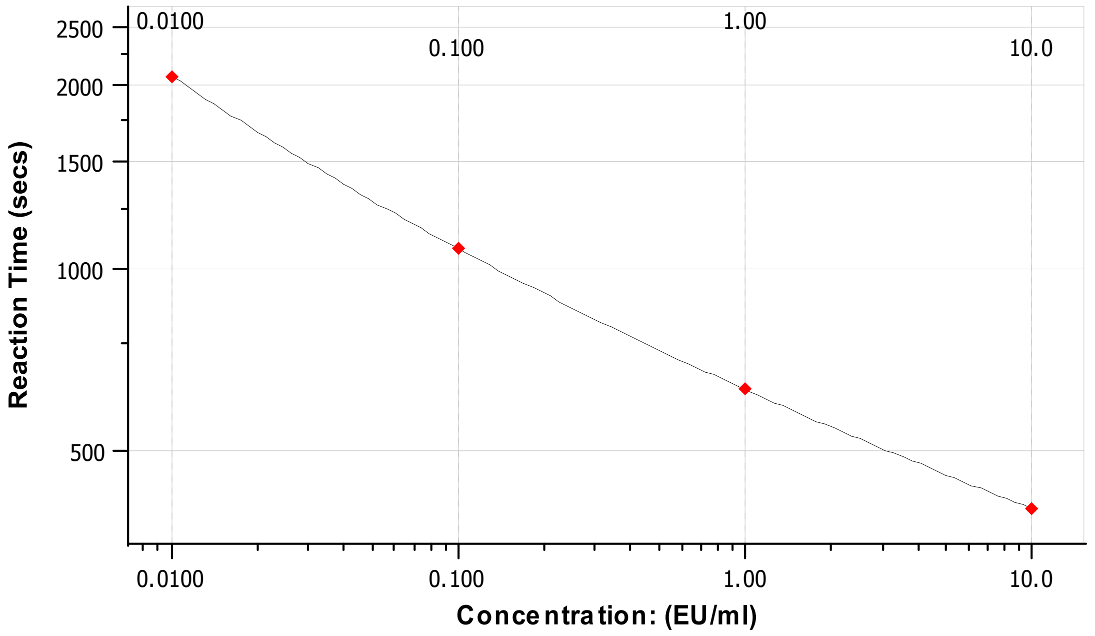

Standards Results

Standards	Conc./Dil.	Average Reaction Time (sec)	Back Prediction (Power Curve)	% CV	Status	Notes
Blank	Blank	****	****	N/A	Undefined	*
Standard 1	0.0100	2078	0.0100	1.19%	PASS	
Standard 2	0.100	1078	0.100	0.66%	PASS	
Standard 3	1.00	631	1.00	2.69%	PASS	
Standard 4	10.0	401	10.0	3.88%	PASS	

Product Results

Samples	Conc./Dil.	Lot Number	Product ID	Results (Power Curve)	%CV	% PPC Recovery	% PPC Rec. Status	PPC %CV	Notes
OM-MSC - Antibiotic									
1	40	CS022-01 (1) -	033803	< 0.400		99%	PASS	2.16%	*
OM-MSC - Antibiotic									
1	40	CS022-02 (2) -	033805	< 0.400		121%	PASS	2.75%	*
OM-MSC - Antibiotic									
1	40	CS022-03 (3) -	033807	< 0.400		102%	PASS	1.12%	*

Curve Parameters

Parameter	Value	Specification	Status
Correlation Coefficient	-0.997	-1.000 to -0.980	PASS
Slope	-0.238	-0.400 to -0.100	PASS
Y Intercept	2.820	2.500 to 3.500	PASS

Signed By : *Fiona Mitchell* (et1-sig) Date/Time : 05/10/2022 13:51:03

Fiona Mitchell (F.MITCHELL)

Reviewed By : *Stuart Doig* (et1-sig) Date/Time : 05/10/2022 14:00:56

Stuart Doig (S.DOIG)

(!! = Masked, **** = reaction time > 2100, ???? = atypical, # = Modified, >>>> = High OD, See Comment Page)

(In Notes : ! = Masked Point(s), * = Point(s) Did Not React, ? = Atypical Point(s), # = Modified, > = High OD, <LS = Less than the lowest standard)

Performed By : F.MITCHELL	LAL Lot No. : XL041KRP2Y	Exp : 02/04/2023	Time : 12:11:25
PYROGENT-5000 - Routine Test	Water Lot No. : 0001098994	Exp : 10/03/2024	Date : 05/10/2022
Template : 051022/01	Endotoxin Lot No. : 0001011300	Exp : 02/06/2025	ELx808
Temperature : 36.9°C - 37.0°C	Recon. Buffer Lot No. : 0001019067	Exp : 01/07/2023	S/N 253635

Linear Regression : Correlation Coefficient = -0.997 Slope = -0.238 Y-Intercept = 2.820
PowerCurve : A = 2.8000 B = -0.2119 C = 0.0179 D = -0.0028 E = 0.0000

Product AER

Dil./Conc.	Parameter	Results (Power Curve)	Specification	Status	Notes
		**OM-MSC - Antibiotic — Lot No. CS022-01 (1) - ID. 033803**			
40	%CV	N/A	< 10%	Undefined	*<LS or Undefined
	Endotoxin	< 0.400 EU/ml		N/A	
PPC	%CV	2.16%	< 10%	PASS	
PPC	% PPC Recovery	99%	50 to 200	PASS	
		**OM-MSC - Antibiotic — Lot No. CS022-02 (2) - ID. 033805**			
40	%CV	N/A	< 10%	Undefined	*<LS or Undefined
	Endotoxin	< 0.400 EU/ml		N/A	
PPC	%CV	2.75%	< 10%	PASS	
PPC	% PPC Recovery	121%	50 to 200	PASS	
		**OM-MSC - Antibiotic — Lot No. CS022-03 (3) - ID. 033807**			
40	%CV	N/A	< 10%	Undefined	*<LS or Undefined
	Endotoxin	< 0.400 EU/ml		N/A	
PPC	%CV	1.12%	< 10%	PASS	
PPC	% PPC Recovery	102%	50 to 200	PASS	

Reader Parameters: Delta t (s) = 60 Measurement Filter (nm) = 340 Delta mOD = 30 Actual/Max Reads = 36/100

Accessories Summary

Name	Lot No.	Manufacturer	Cal. Due Date	Exp
200ul Pipette Tip	L131916G	Star Lab		31/12/2026
5ml Sample Tube	05322005	Corning		28/02/2027
ClipTip Pipette Tips	20350B0	Thermo Scientific		31/12/2025
Costar 96 Well Plate	25721035	Corning		13/09/2024
F1 ClipTip 8 Channel	OH51795	Thermo Scientific	26/03/2023	
Finnpipette 100-1000	CH83006	Thermo Electron Corp	23/03/2023	
Finnpipette 30-300	CH95841	Thermo Electron Corp	23/03/2023	
Finnpipette5-50	CH14831	Thermo Scientific	23/03/2023	
Microplate Reader	253635	Lonza	31/03/2023	31/03/2023
Pipet Tips 1250uL	222269	VWR		30/09/2025
Reagent Reservoirs	518741	SLS		15/07/2025

Log Summary

Description (Comment)	User ID	Date
Routine Test RunDateTime: 05/10/2022 12:11:26 (GMT Daylight Time) Serial # : 253635	F.MITCHELL	05/10/2022 12:11:25
Analyst E-sig applied - F.MITCHELL Run Date/Time - Oct 05 2022 12:11:25 PM (GMT Daylight Time) Number - 0	F.MITCHELL	05/10/2022 13:51:03
Reviewer E-sig applied - S.DOIG Run Date/Time - Oct 05 2022 12:11:25 PM (GMT Daylight Time) Number - 0	S.DOIG	05/10/2022 14:00:56

Signed By : *Fiona Mitchell* (et1-sig) Date/Time : 05/10/2022 13:51:03
Fiona Mitchell (F.MITCHELL)

Reviewed By : *Stuart Doig* (et1-sig) Date/Time : 05/10/2022 14:00:56
Stuart Doig (S.DOIG)

(!! = Masked, **** = reaction time > 2100, ???? = atypical, # = Modified, >>>> = High OD, See Comment Page)
(In Notes : ! = Masked Point(s), * = Point(s) Did Not React, ? = Atypical Point(s), # = Modified, > = High OD, <LS = Less than the lowest standard)
